# Online discussions about cancer and fertility: an analysis of Reddit threads

**DOI:** 10.1007/s10815-025-03527-0

**Published:** 2025-05-28

**Authors:** Brooke Cherven, Kayla D. Fitch, Eline Nijeboer, James L. Klosky, Vicky Lehmann

**Affiliations:** 1 Aflac Cancer and Blood Disorders Center at Children’s Healthcare of Atlanta, Atlanta, GA USA; 2 Department of Pediatrics, Emory University School of Medicine, Atlanta, GA USA; 3 Wellstar Health System, Marietta, GA USA; 4 Department of Medical Psychology, Amsterdam UMC location University of Amsterdam, Amsterdam, The Netherlands; 5 Cancer Center Amsterdam, Amsterdam, The Netherlands

**Keywords:** Young adults, Cancer, Fertility, Online discussions, Parenthood, Gonadotoxic

## Abstract

**Background:**

Young patients may search the Internet and consult online discussion platforms for health-related information. This can be useful and supportive, but also problematic if misinformation is spread. Fertility-related information in the context of cancer is complex and confounded by uncertainty, which can cause misunderstandings and unnecessary burden.

**Methods:**

Discussions on the online platform Reddit were searched and analyzed for questions around cancer and fertility. A mix of structured coding (e.g., number/types of questions) and qualitative analyses of user questions and comments were used to uncover salient content and interactions online.

**Results:**

A total of *N* = 149 relevant threads were identified, posted on three subreddits related to cancer and fertility. Posted questions aimed at seeking either information (57.7%, *n* = 86) or advice/support (42.3%, *n* = 63). Information-related questions focused on medical aspects (e.g., fertility status, assisted reproductive technologies [ART]), financial aspects (e.g., health insurance), or medical decision-making (e.g., ART, fertility assessments). Advice-related questions focused on the emotional impact of (possible) infertility (e.g., coping, burden of unsuccessful pregnancy attempts). Analyses of *n* = 20 comment sections revealed six themes within answers to information-related questions (e.g., personal experiences/stories to provide advice, offering explanations/ suggestions). These interactions typically occurred in a respectful and supportive tone of voice. While misinformation was infrequent, users sometimes derailed into subdiscussions unrelated to the initially posted question.

**Conclusion:**

Online communities like Reddit offer a place where cancer patients/survivors may seek information and exchange ideas regarding their concerns in real time. Frequent topics of discussion can serve as areas of priority for developing educational and communication interventions in clinical care.

Young adulthood is a life phase that includes various developmental tasks, including the decision of whether or not to become a parent [1]. Being treated for cancer during this time can critically affect possible parenthood [2]. Gonadotoxic cancer treatments like alkylating chemotherapy, radiation or surgery affecting the gonads, and hematopoietic cell transplant [3, 4] impair fertility. The extent of an individual’s infertility risks is further determined by the type of cancer/tumor location and age at diagnosis. Infertility rates in young cancer survivors range between 10 and 60% or can be well above 90% after hematopoietic cell transplant [5–8]. Thus, patients’ future fertility can be highly uncertain. Moreover, adjuvant hormone therapies following initial cancer therapy can also impair fertility as long as these are administered, which typically occurs over several years [9]. For many, cancer treatments not only physically impair fertility but also delay their ability to start a family due to prolonged treatment timelines.

Fertility preservation (e.g., freezing sperm, oocytes, embryos, or ovarian tissue) before gonadotoxic treatments enables patients to potentially use assisted reproductive technologies (e.g., in-vitro fertilization [IVF]) later in life [10–13]). However, barriers to fertility preservation include cost and urgency to initiate cancer treatment [14, 15]. The complexity and uncertainties of infertility risks *and* fertility preservation can be difficult to grasp for patients at a time when they may be preoccupied with their disease and survival [16, 17], leading to concerns, misperceptions (e.g., too optimistic or pessimistic views on their ability to have children), and unmet informational needs [18–23]. Moreover, oncologists tend to neglect discussing fertility with patients who are single, female, less educated, identify as sexual and/or gender minority, or already have children [24–26], which can create healthcare inequalities. After treatment, survivors experience continued uncertainty regarding fertility, as well as fertility-related distress [27–29].

All of the above may encourage patients to seek (more) information and support online. Consulting the Internet for health-related information and emotional support can be helpful, but peer-to-peer discussion platforms may be prone to spreading misinterpreted or incorrect information [30]. The accuracy of peer-to-peer discussions around cancer and fertility is unknown, and the literacy of users to identify misinformation likely varies. People with lower health literacy tend to consult social media and trust blogs or webpages of celebrities/influencers more than professional medical websites [31], which increases the risk of consuming misinformation. At the same time, the Internet may also offer a space for patients to openly share, support, and discuss sensitive information anonymously. Thus, online discussion platforms can provide crucial insights into the information needs and understanding of fertility of young adults with cancer. In turn, such insights can be utilized to improve information provision in clinical practice and online.

This study aimed to examine which fertility-related *questions* people post online when being faced with cancer to enhance insights into patient understanding of fertility problems. Subsequent comments and answers were analyzed to assess what information was discussed, how users interacted within the comment section, and whether misinformation was shared.

## Methods

### Online search

Data were collected from the open-source online discussion platform *Reddit* with more than 52 million daily users worldwide. Users can scroll through popular content and/or follow certain groups (so-called “subreddits”) based on interests (e.g., sports, science, cooking). In the Reddit community, people are referred to as “users” and a post is called a “thread.” Users who author an initial thread are considered “original poster” (OP). For this project, three subreddits were screened: r/cancer (55,900 subscribers), r/infertility (42,500 subscribers), and r/TryingForABaby (105,300 subscribers by the time we started planning our data scraping: November 2023). A search strategy was developed, including search terms related to cancer and its treatment, fertility, pregnancy, and/or cryopreservation (Table 1). The selected subreddits were screened using the statistical software *R* (https://www.r-project.org/) using the *Reddit Extracto_R* package and data scraping was completed in January 2024. Identified content was downloaded into Excel files (including initial post, number of comments, URL, and time of posting).

**Table 1:** Search terms used to scrape information on the three examined sub-Reddits

Subreddit	Search terms
r/cancer	preserv OR cryo OR freezing OR froze OR bank OR sperm OR semen OR oocytes OR eggs OR embryo OR tissue OR IVF OR ICSI OR IUI OR insemination OR ART OR miscarriage OR baby OR pregnant OR pregnancy OR embryo OR donor OR fertile OR infertile OR sterile OR infertility OR fertility OR sterility OR subfertile OR fertilization OR sterilization
r/tryingForABaby	cancer OR tumor OR tumour OR oncology OR leukemia OR leukaemia OR lymphoma OR Hodgkin OR non-hodgkin OR NHL OR sarcoma OR chemo OR radiation OR radiotherapy OR BMT OR HSCT OR oncologist
r/infertility	cancer OR tumor OR tumour OR oncology OR leukemia OR leukaemia OR lymphoma OR Hodgkin OR non-hodgkin OR NHL OR sarcoma OR chemo OR radiation OR radiotherapy OR BMT OR HSCT OR oncologist

According to Reddit, the majority of their users are aged 18–35 years, representing the target audience for this study. Data collections for research through Reddit are increasing [32], while usability and validity have been demonstrated [33]. The free usage of publicly available online data is thoroughly discussed [32] and Reddit allows the usage of their data for ethical non-commercial research purposes (https://redditinc.com/policies/user-agreement). Similar to other qualitative studies on cancer using Reddit data [34, 35] and in further respecting the privacy of users, no user data (e.g., user names, protected health data) were analyzed and presented quotes were shortened. This study was approved and ruled as exempt from in-depth medical review by the Medical Ethical Committee of the Amsterdam UMC (2003.0607).

Threads were included if they contained (1) one or more questions (i.e., in order to elicit answers/interaction and representing information seeking behavior). Such questions had to be (2) related to fertility in the broadest sense (e.g., regarding fertility risks, fertility status, fertility preservation, family planning, ART) and (3) refer to the situation of cancer (based on content and/or posted by users who identified as a cancer patient or survivor or a partner of a cancer patient/survivor).

### Analyses

First, all identified threads were screened by two coders (EN, VL) for fertility-related questions in the context of cancer. Thereby, both coders familiarized themselves with the data, and a preliminary coding scheme was developed. Second, relevant threads (about fertility and cancer) were divided among authors and double-coded (BC, KDF, EN, VL) for their *type of question* (information vs. advice/support) and *content* (medical aspects, treatment decisions, family building, emotional impact, dating/relationships, financial/work-related aspects, or alternative family building) based on the preliminary coding scheme. If relevant, each thread could be given more than one code. Possible subthemes were added as needed to further label the questions’ content in a bottom-up manner. Any inconsistencies or doubts were discussed among all authors. Third, of all threads with an information-related question, the 20 threads with the highest number of comments (i.e., those eliciting the most discussion and therefore potentially spreading misinformation) were selected for analysis of the comment section. Comments of these 20 threads were analyzed using content analyses (i.e., identifying reoccurring salient themes). Thereby, we also examined how users interacted with one another (e.g., whether new topics were introduced). Posts were reviewed by all authors to identify possible misinformation (i.e., statements known to be false) and evaluate the accuracy of medical recommendations by users (e.g., if the recommendations align with standard procedures of care).

## Results

### Original posters’ (OP) questions

**Fig. 1:**
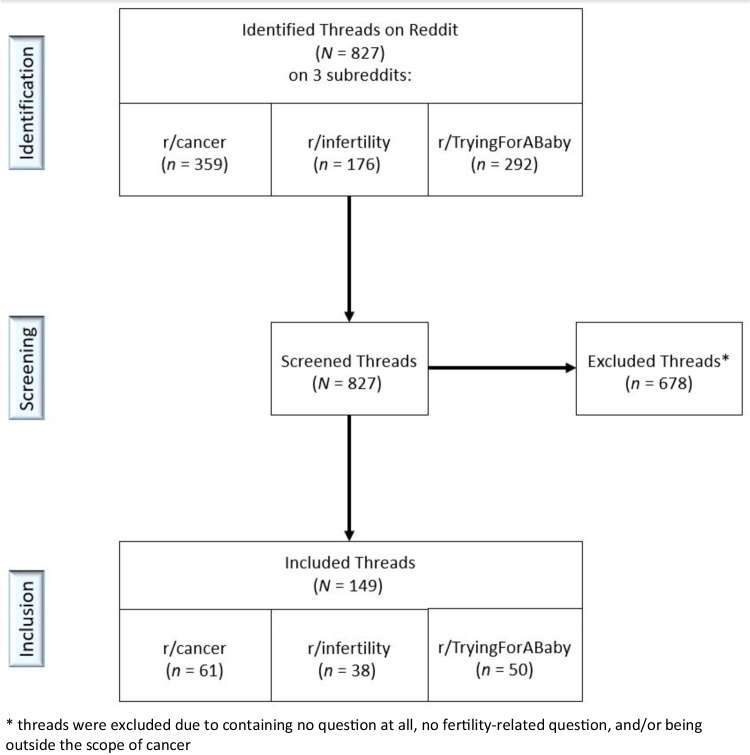
Flow chart of identified and included Reddit threads

A total of *N* = 149 threads contained fertility-related questions and were included (see flow chart in Fig. 1). These threads were posted on Reddit online between 2019 and 2024. Most threads were posted by users who described themselves as cancer patients or survivors (71.8%, *n* = 107), whereas 27.5% (*n* = 41) of threads were posted by users who identified as partners of patients/survivors, and *n* = 1 thread contained a generic fertility-related question where the user did not specify their status. Almost two-thirds of questions pertained to female fertility (61.1%, *n* = 91), whereas 36.9% (*n* = 55) pertained to male fertility, and 2.0% (*n* = 3) were generic fertility-related questions. Based on the content, questions were geared toward two goals: seeking (A) fertility-related information (57.7%,* n* = 86) or seeking (B) advice/support (42.3%, *n* = 63).

Questions aimed at eliciting (A) *fertility-related information* focused on requesting facts regarding (A.1) medical aspects (*n* = 64; e.g., fertility status, ART), (A.2) financial aspects (*n* = 10; e.g., health insurance), or (A.3) medical decision-making (*n* = 9; e.g., ART, fertility assessments; Table 2).

**Table 2:** Overview of the coded primary focus of fertility-related questions, separated by whether users were (A) seeking information or (B) advice/emotional support

Question Topic	Information (*n* = 86)	Advice/support (*n* = 63)
Medical aspects	*n* = 64	*n* = 14
	- Fertility status/ effects of treatment on fertility (*n* = 39) - ART (*n* = 11) - Fertility preservation (*n* = 8) - Fertility sparing (*n* = 2) - Increasing chances to conceive (*n* = 2)	- Fertility status/ effects of treatment on fertility (*n* = 11) - ART (*n* = 3)
Financial/ work-related aspects	*n* = 10	*n* = 1
	- Health insurance (*n* = 8) - Sick leave (*n* = 1) - Disclosure at work (*n* = 1)	- Burden and advice on cryopreservation storage (*n* = 1)
Treatment-related decisions/choices	*n* = 9	*n* = 8
	- Cancer treatment (*n* = 1) - Fertility preservation (*n* = 2) - Fertility testing (*n* = 2) - ART (*n* = 4)	- Cancer treatment, sparing (*n* = 4) - Preservation options (*n* = 2) - Fertility testing (*n* = 1) - ART (*n* = 1)
Emotional impact	*n* = 1	*n* = 25
	- Seeking information/ resources specifically for survivors who are pregnant (n = 1)	- Coping with possible infertility (*n* = 16) - Burden of not getting pregnant (*n* = 4) - Feelings that body has failed (*n* = 2) - Seeking similar stories (*n* = 2) - Worries about cryopreserved materials (*n* = 1)
Family building	*n* = 2	*n* = 12
	- information about adoption/ alternatives to biological parenthood (*n* = 2)	- Second-guessing reproductive goals/ Having children when having an uncertain prognosis (*n* = 7) - (not) considering alternatives to biological parenthood (*n* = 4) - Pressure from extended family (*n* = 1)
Dating	-	*n* = 3
		- How to date when infertile (*n* = 3)

Questions about (A.1) medical aspects varied in how broad versus specific they were. For example, *“How often do people really lose fertility from [cancer] treatment? Is it really that high?”* versus *“I take my last chemo tomorrow. I was wondering, when should I go get my hormones and fertility tested? Is there a certain time I have to wait for it to be accurate?”* Example questions for (A.2) financial or work-related aspects included “*If I tell HR my situation* [starting post-treatment IVF] *will they share it with my boss*?” or *“I’m wondering if someone could share the total amount of time and money it took to successfully conceive using a donor?”* An example related to (A.3) medical decision-making was “*if you’ve taken Xeloda, did you take Lupron or anything to help protect your fertility? I was given Lupron throughout IV chemo and asked my oncologist if I should have Lupron with this Xeloda regimen and he said I didn’t need to.*”

Questions aimed at seeking (B) *fertility-related advice/support* focused on the (B.1) emotional impact of fertility problems (*n* = 27; e.g., coping with possible infertility, burden of unsuccessful pregnancy attempts), (B.2) medical aspects (*n* = 14; e.g., worries about fertility), or (B.3) family building (*n* = 10; e.g., doubts when having an uncertain prognosis; Table 2).

When seeking advice/support regarding the (B.1) emotional impact, OPs often provided background information about their current situation, followed by asking whether others had similar experiences (e.g., “*I wanted to see if anyone had the same feeling/experience?/How do you all cope? […] Are you open about your struggles or worries with family/friends?*”). Examples of advice-seeking questions related to (B.2) medical aspects were often aimed at ART procedures, such as: *“three vials of sperm. I don’t know if this is a lot or not […] I have contacted the clinic for more info but I wanted to hear of some personal experiences with ICSI.”* Another OP stated: *“navigating the embryo transfer process and going into this knowing that I will be unable to breastfeed. This has already been an emotional rollercoaster and I constantly feel like I’m missing something. Any advice, practical or emotional, would be greatly appreciated.”* Examples for seeking advice regarding (B.3) family building included *“I need to look into surrogacy but I don’t know where to start […]. If you can offer any guidance I would really appreciate your help”* as well as someone with a partner with advanced cancer: “*The idea of being a single parent terrifies me. But we both really want to have a child […] I would love some perspective from those [who] have kids or are planning kids.”*

If OPs posted several questions in one thread, which pertained to the same topic (e.g., emotional aspects as quoted above), these questions were coded once. However, in 34 out of 149 threads (22.8%), OPs posted several questions aimed at multiple topics. These included, for example, seeking information about medical aspects, followed by asking for advice regarding the emotional impact or decision-making.

### User comments

Of threads that aimed at seeking *fertility-related information*, the 20 threads with the highest number of comments were analyzed, which ranged from 18 to 57 comments. OPs were part of an average of 26% of these comments (range 0–42%), thanking users for their input, specifying follow-up questions, or consoling others who shared similar experiences. Based on the analyzed threads, the following six categories in user reactions and interactions were identified (see examples in Table 3).

**Table 3:** User interaction categories identified in the *n* = 20 comment sections of information-related fertility questions

	Interaction category	Description/examples
(A)	Answering with personal experiences	- establish common grounds: “*personally, I would* …” / “… *but it’s personal*.“
(B)	Clarification and agreement	- following-up for clarification (e.g., *“Do you have short-term disability through your employer?”*) - treatment-related steps/ issues were typically in alignment across comments
(C)	Explanations and suggestions	- stating facts, often without backup/reference - recommending alternatives (e.g., fertility sparing; ways of coping) “*My doctors tried to get me to freeze my eggs before chemo […] they told me it would be about $7,000. […] As a 26 year old I decided that I was not willing to pay that much so they opted for Lupron injections […] It shuts down your ovaries and makes your body think you are in menopause to protect your eggs and ovaries as much as possible. I would ask your doctors about this option!*”
(D)	Tone of voice	- respectful and supportive - recommend talking to providers - at times blunt: “*maybe you need to change your expectations a bit then. Recurrence is a part of survivorship. Maybe the biggest part.”* [OP’s partner, a cancer survivor, was reluctant to complete semen analysis; commenter expressed little understanding: “Look, I get it. It can be a touchy subject for guys. […] But there’s a not insignificant chance that his cancer treatments did have a lasting effect. So what does he have to do? J*rk off into a cup. […] Know what [women] have to do when we need testing? [providing details] So, honestly, I don’t really care if my husband found it awkward […] He can handle his part.”
(E)	Language use	- shorter if threads were about male factor infertility - common use of abbreviations without explanations, such as medical terms (HSG, IVF) or fertility-related aspects (*TTC*, *BFP*) - occasionally correcting language (e.g., use “*unassisted*” instead of “*natural*” attempts to conceive)
(F)	Derailing and discussions	- sub-discussions unrelated to OPs’ questions

(A) Answering with personal experiences

Users often commented by disclosing their own personal experiences to either establish a common ground of shared understanding with the OP or to underline why they would or did make certain decisions. If OP’s questions referred to certain steps in the process of conceiving/ART, users often shared clear preferences backed by their own experiences.

(B) Clarifications and Aareement

Users also asked follow-up questions to gain more specific insights, for example, about semen analysis results or number of frozen vials. Yet, users typically recommended one option/step over another. Thus, different users’ recommendations aligned (e.g., try intrauterine insemination before IVF, hysterosalpingography (HSG) before IVF) with very few users advocating for an opposite idea/route.

(C) Explanations and suggestions

Users also provided direct or clear explanations to OPs’ questions. These were often stated as facts without further background information or references. In rare instances, users corroborated their comments by saying they were a physician/medical professional or working in human resources (e.g., for threads related to medical questions or finances/work respectively).

Sometimes, comments focused on providing alternatives or solutions/suggestions for OPs to consider (e.g., coping with certain side effects). In one example, OP questioned whether they should delay cancer treatment to complete fertility preservation, and many comments offered alternative options (e.g., recommending “*Lupron injections*” during cancer treatment, “*ovarian transposition*,” “*ovarian tissue freezing*,” or “*adoption*”). Yet, whether these were realistic or feasible options for the OP remains unclear. Users sometimes included links to websites for more information, suggested to follow certain individuals on Instagram, or to join Facebook groups for emotional support.

(D) Tone of voice

The overall tone of users and conversations was primarily respectful and supportive, for which OPs appeared appreciative. In few instances, users provided more blunt and candid opinions. Thereby, the greater physical and mental burden of ART for women was emphasized candidly and sometimes, humor was used. In one thread, where an OP expressed questions and devastation about entering menopause after pelvic radiation and chemotherapy, many users were rather astonished that providers had not offered fertility preservation prior to treatment and commented negatively about the care team. In other instances, users reassured OPs that providers’ recommendations were reasonable. For some specific situations (e.g., conception and chemotherapy; fertility status), users recommended that OPs should talk to their providers, while also raising ethical questions, in a thoughtful/respectful manner, for example, about whether one should try to have children in certain situations (e.g., advanced cancer).

(E) Language use

Answers to threads about male factor infertility and/or threads by OPs who identified as male were rather short and more factual instead of focusing on emotional aspects or sharing users’ own experiences to answer questions.

Users also commented on OPs’ language use and insisted on edits (e.g., using “*unassisted*” instead of “*natural*” attempts to conceive).

(F) Derailing and discussing

At times, answers to threads derailed into short sub-discussions between users. Such sub-discussions were unrelated to OPs’ questions, but instead included follow-up questions to other users’ comments, opinions, elaborations on own stories, or wishing each other well. Such conversations also frequently tapped into how people coped with their cancer journey.

Given that users were often in agreement or provided their suggestions as options to consider, few corrections were expressed amongst users. Yet, one thread asking about legal issues (i.e., HR and sick leave) led to one user insisting on being right, while others reacted and disagreed with this user and were supportive toward OP (i.e., encouragement to tell HR about their situation). Eventually, a user claiming to work in HR also chimed in, providing nuance to previous statements, while another also highlighted that users were unaware of OP’s location and legal situation and suggested that OP should seek local support.

### Accuracy of information

All analyzed comment sections contained primarily accurate information, but misunderstandings (e.g., due to missing information/context) occurred. This prevented users from getting a clear picture initially, but it was resolved in all threads as users and OPs typically shared additional information or corrected one another (e.g., above HR example). In another instance, an OP (who identified as female and newly diagnosed with cancer) was concerned about not having enough time to freeze eggs before starting chemo in 3 weeks and asked for others’ experiences/suggestions. One commenter replied: *“That’s definitely not normally a three week process. In our case I had to give [wife] shots for about eight weeks leading up to the egg retrieval.*” The timeline of 8 weeks is typically not applicable for fertility preservation prior to cancer treatment, which was clarified by another user: “I*’m an OBGYN and *[... it]*, usually takes 3–4 weeks for the whole process.”* Another example included a thread where an OP shared frustration about a fertility clinic’s policy to complete sexually transmitted infection (STI) testing as a male partner prior to IVF. Users provided thoughtful comments about this very common policy and accurately described underlying reasons (e.g., risk of complication if women who pursue IVF have STIs). OP later expressed that they had omitted a crucial detail (i.e., living in abstinence), which provided more context for their frustration.

While not inaccurate, some comments included experiences of success that may raise unrealistic expectations for OPs. For example, an OP who identified as a female survivor posted about starting letrozole to aid in conceiving but shared that she will not be able to afford intrauterine insemination (IUI) and asked for experiences or suggestions from the community. One user commented that her “*best friend did one round of letrozole without IUI and delivered healthy twins”* and another shared *“Yes it worked first time!!”* These comments may provide the OP with hope and encouragement but also may result in distress if she does not have a similar experience.

In another instance, OP used the term IVF but was describing fertility preservation prior to cancer treatment. This may pose risks for misunderstanding, but it appeared that user replies were not misleading/false and that users correctly identified the concept being discussed.

## Discussion

Through the rather novel approach of using existing discussions on Reddit, we identified possible *information* needs (e.g., medical aspects, finances/work, decision-making) and needs for *advice/support* (e.g., emotional impact of fertility problems, worries, family building) in the light of cancer at reproductive age. We further extend prior work by analyzing the comment sections to better understand the types of feedback provided to these needs/questions, how users interact, and whether misinformation was spread.

Reddit users’ fertility-related questions focused on the impact of cancer treatment on fertility, ART, or other medical aspects related to fertility. This is unsurprising given that previous research has identified that cancer patients are often not offered fertility counseling from specialists, few undergo preservation [18, 36], and feeling ill-informed is common in survivorship [27, 29, 37]. Financial and work-related questions represent another known area of concern for patients when considering fertility preservation and/or family building [38]. Importantly, OPs’ questions also focused on the social, emotional, relationship, and partner impact of potential infertility and family building, topics that are rarely addressed in the cancer setting. While fertility-related information and resources are available through various websites, most have been found to lack comprehensiveness and appeal for cancer patients/survivors [39] and many healthcare professionals are reluctant to use social media to provide reproductive health information [40]. Thus, online communities like Reddit offer an alternative place where patients/survivors can seek information and exchange ideas regarding their concerns at any time and at their own pace. The fertility-related questions identified in this study can also be directly translated to inform patient support platforms, social support networks, and interventions.

Our analysis of the comment sections focused on information-related questions (as opposed to advice/support) to assess whether misinformation was spread. We found that most commenters were emotionally supportive regardless, underscoring the help users can find in online communities. Users often shared their own experiences in response to OPs’ questions, which can create a sense of shared understanding and a community where people with cancer feel comfortable seeking information. Notably, it is impossible to verify the authenticity of online discussions, but OPs sometimes explicitly stated that they enjoyed hearing other’s experiences as part of their information seeking process. Yet, sometimes commenters used words that triggered OPs and increased their distress (e.g., cancer recurrence; Table 3).

We did not identify a large amount of misinformation shared within comments, and in some cases, users corrected each other when misinformation or possible misinterpretations were shared. Occasionally, commenters identified as professionals relevant to OPs’ question (e.g., medicine, HR) and provided well-informed advice. Nevertheless, the utility of information shared through comments may vary. For example, OPs are often not able to provide the entire context and nuances for their individual situations (e.g., clinical, relational, or financial considerations). Therefore, the advice shared through comments may not be feasible or applicable to specific situations (e.g., certain types of drugs, local legislation). Nevertheless, for some users, this may provide an opportunity to explore options they had not considered before and which they could discuss with their providers. However, for others, some comments may increase distress and confusion. For example, when users shared surprise about an OP not being offered fertility preservation, it could increase distress/regret for the OP. Yet, it could also encourage OP to discuss reasons why no preservation was offered, which ultimately may be beneficial for OP’s understanding. Other suggestions may only be relevant for specific cases, such as the use of gonadotropin-releasing hormone agonists which are currently only recommended as a fertility-sparing approach for females with breast cancer as their efficacy in other cancer diagnoses is unclear [10]. In fact, oncofertility is a highly complex field and requires individual tailoring [41], which many commenters reinforced by recommending the OP should talk to their own providers. Users who are seeking information online may find these discussions helpful as a springboard for considering fertility-related issues, which are best followed by discussion with providers who are familiar with the OPs’ clinical scenario [34]. In short, commenters did not share misinformation per se, but the information shared is not necessarily relevant to each individual OP and their initial questions.

### Limitations

We utilized existing online discussions, which had the advantage of not burdening human subjects by participating in research and assessing emotionally burdensome concepts (i.e., infertility). This approach was also intended to include experiences of patients who are often not well represented in research (e.g., those with lower health literacy, lower education), given that fertility‐related knowledge is traditionally studied through surveys (potentially over‐representing female and highly educated people). With our approach, we are unable to confirm whether we indeed included these people (i.e., user demographics are unavailable), but using real-life data increases the ecological validity of our findings. Our approach also limits our ability to determine informational needs associated with specific background characteristics (e.g., age groups or type of cancer diagnoses), while we are also unable to verify whether users were cancer patients or survivors. Nevertheless, Reddit users are typically in our targeted range of reproductive-aged people [42] and included threads self-disclosed a variety of types of cancer, further substantiating our findings. Yet, our results may not reflect experiences of non-English speaking people while overrepresenting the experiences of people from the USA. The Reddit community is obviously limited to users who have access to the internet, but it is free to use, which increased the likelihood that users come from diverse socioeconomic backgrounds. Importantly, all questions and comments appeared genuine, and we did not detect bots. Users sometimes suggested external websites, which we did not check for accuracy. Suggestions to follow influencers on Instagram or joining Facebook (peer support) groups may be regarded with hesitation, although emotional support through such fora may be particularly helpful to young patients/survivors, as cancer is relatively rare at that age and connecting with others can be challenging.

## Conclusions and implications

Reddit users sought medical information related to cancer and fertility, treatment options, finances, emotional support, and family building advice. While misinformation was minimal, not all comments were relevant to OPs’ questions, and some had the potential for distress. Yet, it may also encourage patients/survivors to talk to their providers and ask for clarifications, which can be beneficial after all. Given the overall supportive tone and interactions between users, Reddit can be an online space for young people with cancer to connect and seek advice regarding fertility-related issues.

Importantly, questions posed online can serve as direct input for what healthcare providers missed but should have discussed with patients/survivors in clinical care. Information about fertility assessments, support in navigating the process of (un)assisted reproductive options in survivorship, information about insurance coverage, and financial/work-related considerations are all areas that should be addressed through multidisciplinary teams. In further developing future clinical care and research, identified questions and themes from Reddit could be used as a blueprint to develop educational tools and counseling scripts for clinical care. Co-designing such educational materials with patient advocates can bridge the gap between peer-to-peer advice (as found on Reddit) while also ensuring that medical information is accurate. Frequently asked questions on Reddit could be translated into digital resources with expert medical responses and posted on cancer support websites. Recurring concerns highlighted in our findings (e.g., ART, fertility status assessment, finances/insurance) can be proactively addressed during clinical visits and could inform the development of resources for fertility and cancer support organizations. Presented quotes and scenarios can also be included in trainings for oncology and reproductive healthcare providers in preparation for discussions with patients and families. Finally, identified posts and examples which focused on financial stress and insurance coverage can be utilized to advocate for equitable fertility preservation policies at the institutional and national levels.

## Data Availability

Data generated and/or underlying the current study are available from the corresponding author on reasonable request.
